# Predictive Factors for Failed Sentinel Lymph Node Mapping in Endometrial Cancer: A Retrospective Multicenter Study

**DOI:** 10.1002/jso.70106

**Published:** 2025-10-11

**Authors:** Cristina Taliento, Gennaro Scutiero, Giuseppe Cucinella, Vito Chiantera, Giovanni Pontrelli, Marko Klaric, Dragan Belci, Martin Steinkasserer, Stefano Restaino, Ruby Martinello, Orsola Brasile, Martina Arcieri, Sara Pregnolato, Giulia Pellecchia, Giulia Soraci, Rosa Taratufolo, Carmelia Milano, Marcello Desgro, Francesca Davià, Mariano Catello Di Donna, Dino Becic, Sara Notaro, Giuseppe Vizzielli, Pantaleo Greco

**Affiliations:** ^1^ Department of Medical Sciences Obstetrics and Gynecology Unit Ferrara Italy; ^2^ Department of Development and Regeneration KU Leuven Leuven Belgium; ^3^ Gynecologic Oncology Unit Istituto Nazionale Tumori IRCCS Napoli Italy; ^4^ Department of Gynecology and Obstetrics, Hospital of Bolzano (SABES‐ASDAA) Teaching Hospital of Paracelsius Medical University (PMU) Bolzano‐Bozen Italy; ^5^ Clinical Hospital Center Rijeka Clinic for Gynecology and Obstetrics Rijeka Croatia; ^6^ Department of Gynaecology General Hospital Pula Pula Croatia; ^7^ Department of Maternal and Child Health, Obstetrics and Gynecology Clinic Ospedale Santa Maria della Misericordia, Azienda Sanitaria Universitaria Friuli Centrale Udine Italy; ^8^ Department of Medicine (DMED) University of Udine Udine Italy; ^9^ Obstetrics and Gynaecology Clinic, Policlinico Hospital of Abano Terme Padua Italy

**Keywords:** endometrial cancer, failed SLN mapping, ICG injection, predictive factors, sentinel lymph node

## Abstract

**Background and Objectives:**

This study aims to evaluate the predictive factors associated with failed sentinel lymph node (SLN) mapping in a large, retrospective cohort of patients with early‐stage endometrial cancer (EC).

**Methods:**

We retrospectively evaluated a series of EC patients who underwent laparoscopic SLN mapping with intracervical indocyanine green (ICG) injection in five referred oncological centers from January 2019 to March 2024. We compared the clinical and pathological features of bilateral and failed SLN mapping, which was defined as either unilateral mapping or no SLN mapping. Logistic regression was used to identify predictors of failure.

**Results:**

Among 623 analyzed patients, 437 (70.14%) had a successful bilateral procedure. On univariate analysis, age (*p* = 0.03), non‐endometrioid histology (*p* = 0.02) and previous vaginal delivery (*p* = 0.015) were significant associated with failed SLN mapping. On multivariable analysis, only increasing age (OR 1.03; 95% CI, 1.01–1.04, *p* = 0.03) and non‐endometrioid histology (OR 1.81; 95% CI, 1.01–3.19) were independently associated with unsuccessful procedure. No significant differences were observed for BMI, enlarged lymph nodes, intraoperative lysis of adhesion, LVSI, grade 3, and FIGO stage.

**Conclusions:**

Increasing age and non‐endometrioid histology are independent predictors of bilateral SLN mapping failure in EC patients undergoing SLN mapping with cervical ICG injection.

## 1 Introduction

Lymph node metastasis is a well‐established negative prognostic factor in patients with endometrial cancer, guiding treatment decisions towards the need for adjuvant therapy [1, 2, 3]. Therefore, the evaluation of lymph nodes through surgical staging constitutes a standard practice in the surgical management of endometrial cancer. Despite two prospective trials demonstrating no improvement in disease‐free or overall survival in patients with early‐stage endometrial cancer who underwent lymphadenectomy [4, 5], nodal assessment remains crucial for accurate staging and prognostic assessment.

Over the last fifteen years, sentinel lymph node (SLN) mapping has emerged as a safe, feasible, and highly sensitive technique for assessing nodal status in patients with endometrial cancer [6, 7, 8, 9]. With the increasing evidence supporting SLN mapping, this technique has been included in several international guidelines and widely adopted in clinical practice as a valid alternative to lymphadenectomy [1, 10].

SLN mapping is associated with lower morbidity and fewer severe complications compared to pelvic lymphadenectomy, such as lower extremity lymphedema and lymphocysts, which can negatively impact quality of life [11, 12]. Moreover, SLN mapping improved the detection of extrauterine nodal disease, particularly through the implementation of ultrastaging techniques on SLNs, which enabled the identification of low‐volume metastases [13]. Furthermore, a meta‐analysis involving 3,536 patients confirmed that SLN mapping is non‐inferior to lymphadenectomy in detecting paraaortic nodal involvement with equal recurrence rates [14]. Thus, detection of SLN is fundamental since our current approach relies significantly on the identification of only a few nodes [15].

Despite improvements in the technique, failed SLN mapping for endometrial cancer occurs in approximately 21% of cases, with failure defined as either unilateral mapping or the absence of SLN identification in both hemipelvis [16]. According to the widely accepted MSKCC algorithm complete side‐specific pelvic lymphadenectomy should be performed in case of mapping failure [17].

Previous observational studies have attempted to identify factors that predict failed SLN mapping in patients with early‐stage endometrial cancer [18, 19, 20, 21, 22, 23, 24]. However, many of the previous studies have limitations, including small sample sizes, and the use of different tracers for SLN mapping. In a comprehensive meta‐analysis, Raffone et al. identified several key factors associated with mapping failure. These include advanced‐stage disease, an indocyanine green (ICG) dose of less than 3 mL, enlarged lymph nodes, and lymph node involvement with metastasis [16].

Given the importance of accurate bilateral mapping to reduce the need for side‐specific lymphadenectomy, further research involving a larger, homogeneous cohort of patients and standardized methodologies is warranted. Therefore, this study aims to investigate the predictive factors for failed SLN mapping, with the objective of enhancing the technique and improving surgical outcomes for patients with endometrial cancer.

## 2 Methods

This was a retrospective, multicenter, observational study. Patients with a preoperative diagnosis of apparent early‐stage endometrial cancer who underwent surgical staging, including laparoscopic total hysterectomy, bilateral salpingo‐oophorectomy, and SLN mapping with indocyanine green (ICG) injection between January 2019 and March 2024, were included in the study. Data were retrieved from the oncological databases of the following institutions: University Hospital of Ferrara, Italy; National Cancer Institute IRCCS “G. Pascale” Foundation, Napoli, Italy, “Santa Maria della Misericordia” Hospital, Udine, Italy; Clinical Hospital Center Rijeka (CHCR), Croatia; and the General Hospital Pula, Croatia.

The study was approved by the local Ethics Committee of University of Ferrara (CE‐AVEC 7/2024/Oss/AOUFe) and by the Ethics Committee at each participating centers. The protocol of the present study followed the Strengthening Reporting of Observational Studies in Epidemiology (STROBE) statement.

For each SLN mapping procedure, a 25 mg vial with ICG powder was diluted in 20 mL of sterile water. A total of 4 mL of this ICG solution were injected into the cervix at the 3 o′clock and 9 o′clock positions. 1 mL of ICG solution was injected bilaterally, using a small‐caliber needle (22 gage) with penetration to a depth of 1 cm into the stroma, and 1 mL was injected into the submucosal layer. When the SLN was not visualized, patients underwent side‐specific lymphadenectomy; in low‐risk patients—defined as having disease confined to the uterus, FIGO grade 1 or 2 endometrioid histology, and ≤ 50% myometrial invasion ‐ this procedure was omitted [25]. Any enlarged lymph nodes ( ≥ 2 cm) identified during surgical exploration were also resected. In case of either unilateral or no SLN detection, an ipsilateral or bilateral cervical reinjection was performed based on the surgeon's discretion. All participating centers adhered to this standardized protocol.

Demographic, clinical, and surgical data were retrieved for each patient. Patients were finally staged according to the 2009 International Federation of Gynecology and Obstetrics (FIGO) staging system.

The primary outcome assessed was the rate of bilateral SLN mapping failure, defined as unilateral or no SLN mapping.

Statistical analysis was performed using R version 4.2.2 (R Core Team, 2022). Basic descriptive statistics were used to describe the population. Categorical variables were compared using Chi‐Square tests, while continuous variables were analyzed using two‐tailed t‐tests. Univariate logistic regression analyses were performed to identify predictors of failed mapping. The BMI variable was analyzed both as a continuous variable and dichotomized into two categories: obesity (BMI ≥ 30 kg/m²) and severe obesity (BMI ≥ 40 kg/m²). Age was analyzed as a continuous variable to evaluate its association with SLN mapping failure. The effect of age was estimated per 1‐year increase, and results were expressed as odds ratios with corresponding 95% confidence intervals. To evaluate the association between multiple factors and the failure of lymphatic mapping, a multivariable logistic regression analysis was performed. A *p* value of < 0.05 was considered statistically significant.

## 3 Results

Overall, 623 patients with endometrial cancer underwent SLN mapping with intracervical injection of a total of 4 mL of ICG.

The median patient age at diagnosis was 64 years (range: 34–93) with a mean BMI of 28 (range: 17–64). The final FIGO 2009 staging was as follows: IA in 319 (51.2%) patients, IB in 191 (30.66%) patients, II in 37 (5.94%) patients, IIIA in 19 (3.05%) patients, IIIB in 6 (0.96%) patients, IIIC1 in 33 (5.3%) patients, IIIC2 in 9 (1.44%) patients, IVA in 1 (0.16%) patient and IVB in 6 (0.96%) patients. The final tumor grading was FIGO G1 in 224 cases (36.19%), G2 in 280 (45.23%), and G3 in the remaining 114 (18.42%). LVSI (lymph vascular space invasion) was identified in 130 (21%) patients, while myometrial infiltration ≥ 50% was present in 249 (39.9%) patients. Histology was distributed as follows: endometrioid, 567 (91%); serous, 36 (5.78%); clear cell, 11 (1.77%); carcinosarcoma, 1 (0.16%); neuroendocrine, 1 (0.16%); undifferentiated, 3 (0.48%); other, 3 (0.48%) of these latter three cases, two were grade 3 and one was grade 1.

Demographic and clinicopathologic tumor characteristics are shown in Table 1.

**Table 1:** Clinicopathologic characteristic.

Clinicopathologic characteristic	No. of patients (%) mean (SD)
Patient age, median (range)	64 (34–93)
BMI ( ≥ 30 kg/m^2^), median (range)	28 (17–64)
Postmenopausal status, *n* (%)	534 (85.71)
Previous pelvic surgeries, *n* (%)	187 (30.31)
Previous cervical surgeries, *n* (%)	14 (2.27)
Vaginal delivery, *n* (%)	456 (73.91)
Cesarean sections, *n* (%)	88 (14.19)
FIGO stage, *n* (%)	
IA	319 (51.2)
IB	191 (30.66)
II	37 (5.94)
IIIA	19 (3.05)
IIIB	6 (0.96)
IIIC1	33 (5.3)
IIIC2	9 (1.44)
IVA	1 (0.16)
IVB	6 (0.96)
FIGO grade, *n* (%)	
1	224 (36.19)
2	280 (45.23)
3	114 (18.42)
Histotype, *n* (%)	
Endometrioid	567 (91.01)
Serous	36 (5.78)
Clear cell	11 (1.77)
Undifferentiated	3 (0.48)
Neuroendocrine	1 (0.16)
Carcinosarcoma	1 (0.16)
other	3 (0.48)
LVSI, *n* (%)	130 (21.00)
Depth of myometrial invasion ( > 50%), *n* (%)	249 (39.97)
Enlarged lymph nodes, *n* (%)	47 (7.54)
Adenomyosis, *n* (%)	96 (15.51)
Uterine dimension (longitudinal diameter),	74 (10–140)
Uterine dimension (antero‐posterior diameter),	50 (14–125)
Uterine dimension (transverse diameter)	40.5 (15–90)

Abbreviations: BMI, body mass index; FIGO, international Federation of Gynecology and Obstetrics; LVSI, lymph vascular space invasion.

Intraoperative findings of enlarged lymph nodes were present in 47 (7.54%) patients. Adenomyosis was found in 96 (15.51%) patients. Previous pelvic surgeries were reported in 187 (30.31%) patients, while 14 (2.27%) had a history of cervical surgeries. Moreover, 164 (26.3%) patients underwent adhesiolysis during the operation. The median uterine dimensions were 74 mm (10–140) for the longitudinal diameter, 50 mm (14–125) for the antero‐posterior diameter, and 40.5 mm (15–90) for the transverse diameter.

A final bilateral SLN (successful mapping) was identified in 437 (70.14%) patients. Of the 186 (29.86%) patients with failed bilateral SLN mapping, 125 (67.20%) underwent lymphadenectomy. The remaining cases were low risk for which lymphadenectomy was omitted.

**Figure 1:**
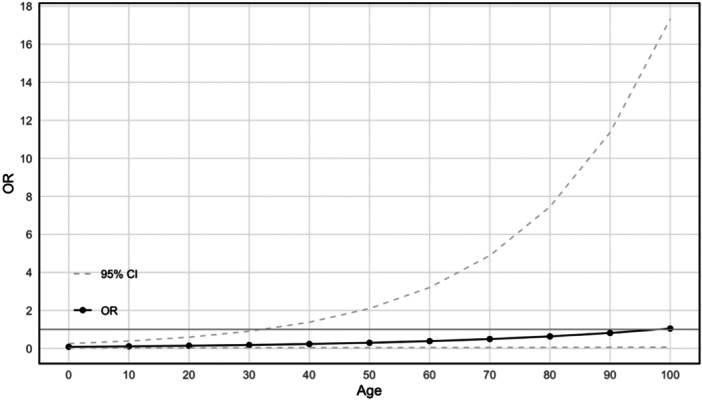
Increasing age. The probability of failed SLN increases by 3% for each additional year of age (OR 1.03; 95% CI, 1.01–1.04 per 1 year increase in age, *p* = 0.03).

Univariate analyses were performed to evaluate the impact of clinical, surgical, and pathological factors on failure to identify a SLN. The association between age (OR 1.03; 95% CI, 1.01–1.04 per 1 year increase in age, *p* = 0.03), non‐endometrioid histology (OR 1.94; 95% CI, 1.09–3.39) and previous vaginal delivery (OR 1.69; 95% CI, 1.12–2.59) was significant associated with SLN mapping failure rate (Table 2). Specifically, we observed that the probability of failed SLN increases by 3% for each additional year of age (Figure 1).

**Table 2:** Univariate analysis of predictors of unsuccessful procedure (unilateral or no SLN mapping).

Characteristic	Overall (*n* = 623)	Bilateral SLN mapping (*n* = 437)	Failed SLN mapping (unilateral or no SLN mapping) (*n* = 186)	Unadjusted OR (95% CI)	*p* value
Age, median (range)	64 (34–93)	63 (34–86)	65 (35–93)	1.03(1.01–1.04 per 1 y increase)	0.004
BMI, kg/m^2^, median (range)	28 (17–64)	28 (18–61)	29 (17–51)	1.00 (0.98–1.03)	0.9513
Obesity (BMI ≥ 30 kg/m2), *n* (%)	254 (41.50%)	172 (40.28%)	82 (44.32%)	1.18 (0.83–1.67)	0.351
Obesity (BMI ≥ 40 kg/m^2^), *n* (%)	47 (7.73%)	35 (8.27%)	12 (6.49%)	0.77 (0.38–1.48)	0.449
Vaginal delivery, *n* (%)	456 (73.91%)	307 (71.06%)	149 (80.54%)	1.69 (1.12–2.59)	0.015
Postmenopausal, *n* (%)	534 (85.71%)	367 (83.98%)	167 (89.78%)	1.68 (0.99–2.95)	0.060
Prior pelvic surgery, *n* (%)	187 (30.31%)	135 (31.11%)	52 (28.42)	0.88 (0.60–1.28)	0.507
Prior cervical surgery, *n* (%)	14 (2.27%)	11 (2.53%)	3 (1.64%)	0.64 (0.14–2.09)	0.501
Previous cesarean Section, *n* (%)	88 (14.19%)	62 (14.25%)	26 (14.05%)	0.98 (0.59–1.60)	0.948
Uterine dimension (longitudinal diameter),	74 (10–140)	75 (10–130)	70 (30–140)	0.99 (0.98–1.01)	0.619
Uterine dimension (antero‐posterior diameter),	50 (14–125)	50 (16–125)	50 (14–90)	0.99 (0.98–1.01)	0.641
Uterine dimension (transverse diameter)	40.5 (15–90)	40 (15–90)	43 (19–90)	1.01 (0.99–1.02)	0.423
Lysis of adhesions, *n* (%)	164 (26.37%)	118 (27.06%)	46 (24.73%)	0.89 (0.59–1.31)	0.546
Enlarged lymph nodes, *n* (%)	47 (7.54%)	33 (7.55%)	14 (7.53%)	0.99 (0.51–1.87)	0.992
Myometrial invasion ≥ 50%, *n* (%)	249 (39.97%)	169 (38.67%)	80 (43.01%)	1.20 (0.84–1.70)	0.312
LVSI, *n* (%)	130 (21.00%)	88 (20.32%)	42 (22.58%)	1.14 (0.75–1.73)	0.527
FIGO stage III (vs I/II), *n* (%)	71 (11.47%)	48 (11.06%)	23 (12.43%)	1.14 (0.66–1.92)	0.624
FIGO grade 3, *n* (%)	114 (18.42%)	73 (16.86%)	41 (22.04%)	1.39 (0.90–2.13)	0.128
Positive lymph nodes, *n* (%)	42 (6.74%)	29 (6.64%)	13 (6.99%)	1.06 (0.54– 2.08)	0.87
Non‐endometrioid histology, *n* (%)	55 (8.84%)	31 (7.11%)	24 (12.90%)	1.94 (1.09–3.39)	0.022
Adenomyosis, *n* (%)	96 (15.51%)	69 (15.94%)	27 (14.52%)	0.90 (0.55–1.44)	0.655

Abbreviations: BMI, body mass index; FIGO, the international Federation of Gynecology and Obstetrics; LVSI, lymph vascular space invasion; OR, odds ratio; SLN, sentinel lymph node.

As shown in Table 3, increasing age (OR 1.03; 95% CI, 1.01–1.04, p = 0.03) and non‐endometrioid histology (OR 1.81; 95% CI, 1.01–3.19) remained significant in the multivariate model.

**Table 3:** Multivariate analysis of predictors of unsuccessful procedure (unilateral or no SNL mapping).

Characteristic	Overall (*n* = 623)	Bilateral SLN mapping (*n* = 437)	Failed SLN mapping (unilateral or no SNL mapping) (*n* = 186)	Unadjusted OR (95% CI)	*p* value
Age, median (range)	64 (34–93)	63 (34–86)	65 (35–93)	1.02(1.01–1.04 per 1 y increase)	0.035
Nulliparous	456 (73.91)	307 (71.06)	149 (80.54)	1.49 (0.97–2.33)	0.071
Non‐endometrioid histology	55 (8.84)	31 (7.11%)	24 (12.90)	1.81 (1.01–3.19)	0.042

Abbreviations: OR, odds ratio; SLN, sentinel lymph node.

## 4 Discussion

### 4.1 Summary of the Main Results

In the current multicenter international study, we found that age, non‐endometrioid histology and parity were significantly associated with SLN mapping failure. However, only increasing age and non‐endometrioid histology were confirmed as independent predictors on the multivariable analysis.

### 4.2 Results in the Context of Published Literature

Previous studies have also assessed several factors that might predict the inability to identify the SLN [18, 19, 20, 21, 22, 23, 24].

In the study by Garrett et al., the authors found that increasing BMI was a risk factor for failed mapping [24]. Similarly, Eriksson et al. reported a significant decrease in successful bilateral mapping rates with increasing BMI ≥ 40 kg/m² for both the ICG (*p* = 0.001) and blue‐dye groups (*p* = 0.041) [23]. Conversely, a meta‐analysis of 55 studies including 4,915 women found that an average patient BMI ≥ 30 kg/m² was not significantly associated with SLN detection rates [7].

In our study, we further analyzed the impact of increasing BMI on SLN mapping success by evaluating two BMI thresholds points, 30 kg/m², used to define obesity, and 40 kg/m², representing severe obesity. In both cases, our results showed no statistically significant correlation between higher BMI and SLN mapping failure. These results align with the findings by Ianieri et al., who also observed no association between obesity (BMI ≥ 30 kg/m²) and mapping failure [18].

Another demographic factor impacting the rate of successful mapping is increasing age. Previous studies indicate that aging‐associated remodeling of the vascular wall leads to decreased lymphatic drainage due to a reduction in muscle cells and nitric oxide levels in aged lymphatic collectors [26]. Additionally, aged lymphatic vessels exhibit thin interruptions in the glycocalyx layer that covers lymphatic endothelial cells, resulting in altered permeability [27]. Therefore, we hypothesized that failure of mapping in older patients could be linked to impaired lymphatic drainage that occurs with aging, similar to the changes that occur in veins and arteries. The association between increasing age and unsuccessful SLN mapping procedure in endometrial cancer patients had been previously reported. Tortorella et al. observed that patients with a failed SLN mapping were more likely to be older, with a mean (SD) age of 67.0 (9.2) vs 63.5 (10.5) years (OR, 1.41 per 10‐year increase in age; 95% CI, 1.08–1.84; p = 0.01) [20]. Similarly, Sozzi et al. reported that older age was associated with a reduced SLN detection on univariate analysis (*p* = 0.0122); however, this correlation was not confirmed on multivariable analysis [21].

In our series, another variable for which statistically significant differences were found in the compared groups on the univariate analysis included the vaginal delivery (71.06% in the SLN failed group vs. 80.54% in the successful group). Surprisingly, patients with a history of at least one vaginal delivery were more likely to have failed procedure compared to nulliparous patients (*p* = 0.015). Only one previous study including 327 endometrial cancer patients, assessed this variable as a predictor of unsuccessful procedure, without reporting any significant association [20]. Nevertheless, from a functional perspective, modifications of lymphatic vessels during pregnancy were historically described in the early 1900s [28]. Cunéo and Marcille observed that, while lymphatic vessels in the nonpregnant uterus were very thin, they exhibited hyperplastic changes during gestation [29]. In addition, recent studies demonstrated that placental cytotrophoblasts stimulate lymphatic growth in vivo and in vitro, triggering pregnancy‐induced decidual lymphangiogenesis [30].

Among pathological characteristics (LVSI, myometrial invasion, non‐endometrioid histology, lymph nodes metastasis), we found that only non‐endometrioid histology was associated with unsuccessful bilateral mapping on multivariable analyses (*p* = 0.042). In a multicenter study by Sozzi et al., LVSI, non‐endometrioid histology, and intra‐operative finding of enlarged lymph nodes were identified as independent risk factors for failed mapping in patients undergoing laparoscopic SLN mapping using ICG tracer [21]. Similar results were found in a large retrospective study, where Garrett et al. identified high risk histology as risk factors for failed mapping [24]. Conversely, Tortorella et al. observed no significant associations between bilateral mapping and tumor pathologic characteristics as tumor diameter, grade, myometrial invasion, histologic type, LVSI, uterine weight, presence of fibroids, or positive lymph nodes [20]. Consistent with these results, in the meta‐analysis performed by Boghurta et al., the non‐endometrioid histology was not statistically significant (*p* = 0.51) [7]. However, the heterogeneity of the included studies in terms of site of injection, tracer and surgical approach limits the generalizability of these findings.

The association between advanced FIGO tumor stage and failure of SLN mapping has also been reported. Body et al. found that advanced FIGO stage correlated with failed bilateral detection (*p* = 0.01) [22]. Moreover, a recent retrospective study demonstrated a decrease in successful bilateral SLN mapping in patients with nodal positivity at final histology (*p* = 0.013) [27]. However, conflicting results were reported in two previous studies [20, 21].

In addition, three studies suggests that the presence of enlarged lymph nodes independently affects the bilateral detection of SLNs [20, 21, 24]. Subsequently, these results were confirmed in the meta‐analysis performed by Raffone et al., although the definition of an ‘enlarged′ lymph node was not clearly defined across the included studies [16]. However, in the present study, neither positive lymph nodes (*p* = 0.87) nor the intraoperative finding of enlarged lymph nodes (*p* = 0.992) proved to be predictive factors for failed mapping.

Although not confirmed by our data, an association between the inability to identify a SLN and nodal disease burden cannot be ruled out; however, nodal positivity at final histology remains of limited value in the pursuit of developing a predictive model based on known preoperative factors.

Among intraoperative factors, lysis of adhesions has been identified as a factor that can impact the bilateral SLN detection rate. In one study, Tortorella et al. found that the lysis of adhesions at the start of surgery can independently affect the bilateral detection of SLNs (adjusted OR, 3.07; 95% CI, 1.56–6.07; *p* = 0.001). One possible explanation is that the increased operative time before SLN identification, combined with the presence of adhesions from prior pelvic surgeries, can hinder lymphatic flow. As a result of these findings, the authors recommended delaying the cervical tracer injection until after the completion of adhesiolysis [20]. Consistent with other studies [16, 21], we did not find any association between the lysis of adhesions and failed SLN mapping. However, our data did not allow us to specify how many cases involved lysis performed before or after the tracer injection, which limits our ability to make a true comparison with previous studies.

Understanding the predictive factors for failed SLN mapping can help integrate these predictors into treatment algorithms for selecting patients who require systematic lymphadenectomy in cases of failed SLN detection. These algorithms could include the use of preoperative molecular classification, including the mismatch repair deficiency (MMR) status. Specifically, for patients with low‐risk tumors (e.g., G1/G2 and myometrial invasion < 50%) but who present with MMRd tumors, the addition of molecular markers could help identify those at increased risk of lymph node metastasis [31]. Further studies are needed to validate this integrated approach and assess its clinical impact in refining SLN mapping algorithms, particularly in cases of failed mapping.

## 5 Conclusions

In our study, we found that increasing age and non‐endometrioid histology were independent predictors of failed SLN mapping in patients with endometrial cancer. A history of vaginal delivery increases the risk of failed mapping, although this variable did not reach statistical significance on the multivariable analysis.

## Author Contributions

Conceptualization: T.C., V.G., G.P., R.S., D.F.; Data curation: T.C., C.G., C.V., K.M., B.D., A.M., P.S., P.eG., S.oG., T.R., M.C., D.M.C., B.D.; Formal analysis: T.C., V.G.; Investigation: T.C., P.G., S.M., N.S.; Methodology: T.C., C.G., G.P.; Project administration: T.C.; Resources: C.V., D.F., T.C.; Software: T.C.; Supervision: C.V., P.G., M.R., N.S., V.G., G.P.; Validation: S.G., C.V., P.G., S.M., D.F.; Visualization: C.G., P.eG.; Writing – original draft: T.C., S.G., D.M.; Writing – review and editing: C.G., R.S., B.O., A.M., V.G., G.P., T.C.

## Conflicts of Interest

The authors declare no conflicts of interest.

## Synopsis

This retrospective multicenter study analyzed 623 endometrial cancer patients who underwent laparoscopic sentinel lymph node (SLN) mapping using intracervical indocyanine green (ICG). The analysis identified increasing age and non‐endometrioid histology as independent predictors of bilateral SLN mapping failure, providing insights into factors that may affect mapping success in clinical practice.

## Data Availability

The data that support the findings of this study are available from the corresponding author upon reasonable request. The datasets generated and analyzed during the current study are not publicly available due to institutional policies and patient confidentiality, but they are available from the corresponding author upon reasonable request.
